# Prevalence and risk factors associated with repeat positive SARS-CoV-2 nucleic acid test results among discharged COVID-19 patients: A protocol for systematic review and meta-analysis

**DOI:** 10.1097/MD.0000000000027933

**Published:** 2021-12-03

**Authors:** Yanru Cui, Jilin Wang, Gaofeng Wang, Xiuguo Xie, Lizhen Tian

**Affiliations:** a First College of Clinical Medicine, Shandong University of Traditional Chinese Medicine, Jinan, China; b College of Acupuncture and Massage, Shandong University of Traditional Chinese Medicine, Jinan, China; c Department of Ophthalmology, Affiliated Hospital of Shandong University of Traditional Chinese Medicine, Jinan, China; d Linzi Traditional Chinese Medicine Hospital, Zibo, Shandong, China.

**Keywords:** coronavirus disease 2019, meta-analysis, prevalence, protocol, repeat positive, risk factors

## Abstract

**Background:**

The COVID-19 (coronavirus disease 2019) pandemic continues to have an immense impact on the world at large. COVID-19 patients who meet the discharge criteria, may subsequently exhibit positive viral RNA test results upon subsequent evaluation. This phenomenon has been a major source of research and public health interest, and poses a major challenge to COVID-19 prevention, treatment, and standardized patient management.

**Methods:**

We will search the PubMed, MEDLINE, Embase, Cochrane Clinical Trials Database, China National Knowledge Infrastructure, Wanfang Database, Chinese Science Journal Database, and China Biology Medicine databases for all studies published as of November 2021. Data will be extracted independently by two researchers according to the eligibility criteria. Finally, RevMan 5.3.0 will be implemented for statistical analyses.

**Results:**

The results of this study will show the prevalence and risk factors associated with repeat positive SARS-CoV-2 nucleic acid test results among discharged COVID-19 patients.

**Conclusions:**

This study will provide a reliable evidence-based for the prevalence and risk factors associated with repeat positive SARS-CoV-2 nucleic acid test results among discharged COVID-19 patients.

**Trial registration number:**

CRD42021272447.

## 1 Introduction

The COVID-19 (coronavirus disease 2019) pandemic continues to have an immense impact on the world at large,^[1,2]^ with over 248,632,918 documented cases and 5,030,334 COVID-19 deaths as of November 4, 2021. Patients suffering from COVID-19 often present with a variety of potentially severe symptoms.^[3,4]^ The ongoing COVID-19 pandemic has, to date, infected hundreds of millions of people throughout the world, resulting in severe disease, death, and long-term health consequences even among a large subset of surviving patients.

COVID-19 patients who meet the discharge criteria of having two consecutive nucleic acid analyses respiratory tract samples that are negative for viral RNA, with at least 24 h between sample collections, may subsequently exhibit positive viral RNA test results upon subsequent evaluation. This phenomenon is often referred to as “re-positivity.”^[5,6]^ Indeed, there have been many reports to date of COVID-19 patients who have, some period of time after recovering from the initial infection, had positive nucleic acid test results.^[7–10]^ Only a subset of these “re-positive” patients exhibit symptoms of disease, but all may have the potential to spread the virus to those around them. This phenomenon has been a major source of research and public health interest, and poses a major challenge to COVID-19 prevention, treatment, and standardized patient management. The causes and frequency of this re-positivity phenomenon remain unclear. As such, there is a clear need for a meta-analysis exploring the true prevalence of such re-positivity in order to provide a foundation for the appropriate post-discharge management of COVID-19 patients.

## 2 Methods

### 2.1 Research registration

This study has been registered in the PROSPERO (CRD42021272447), and will be conducted in accordance with the Preferred Reporting Items for Systematic Reviews and Meta-Analyses criteria.^[11]^

### 2.2 Inclusion criteria

Eligible studies will include epidemiological, cross-sectional, case–control, and cohort studies. Patients that had recovered from a COVID-19 infection will be eligible for inclusion in these analyses, without any restrictions pertaining to patient sex, age, ethnicity, or education. The prevalence of SARS-CoV-2 nucleic acid tests re-positivity will be the primary study outcome, as assessed using odds ratios (ORs) and 95% confidence intervals (CIs).

### 2.3 Exclusion criteria

Studies will be excluded from this analysis if they are reviews, case reports, or animal studies. In addition, studies that only discuss the number of cases exhibiting re-positivity without any corresponding discussion of the total caseload will be excluded.

### 2.4 Search strategy

We will search the PubMed, MEDLINE, Embase, Cochrane Clinical Trials Database, China National Knowledge Infrastructure, Wanfang Database, Chinese Science Journal Database, and China Biology Medicine databases for all studies published as of November 2021. Table 1 details the PubMed search strategy that will be employed for this study, and an identical strategy will be used for all other databases.

**Table 1:** Detailed search strategy in PubMed.

No.	Search terms
#1	COVID-19[MeSH Terms]
#2	SARS-CoV-2[MeSH Terms] OR 2019-nCoV [Title/Abstract] OR coronavirus disease 2019 [Title/Abstract] OR Novel coronavirus [Title/Abstract]
#3	#1 OR #2
#4	reinfection[MeSH Terms]
#5	re-detectable [Title/Abstract] OR recurrence [Title/Abstract] OR retest [Title/Abstract] OR re-positive [Title/Abstract]
#6	#4 OR #5
#7	#3 AND #6

### 2.5 Study selection and data extraction

**Figure 1:**
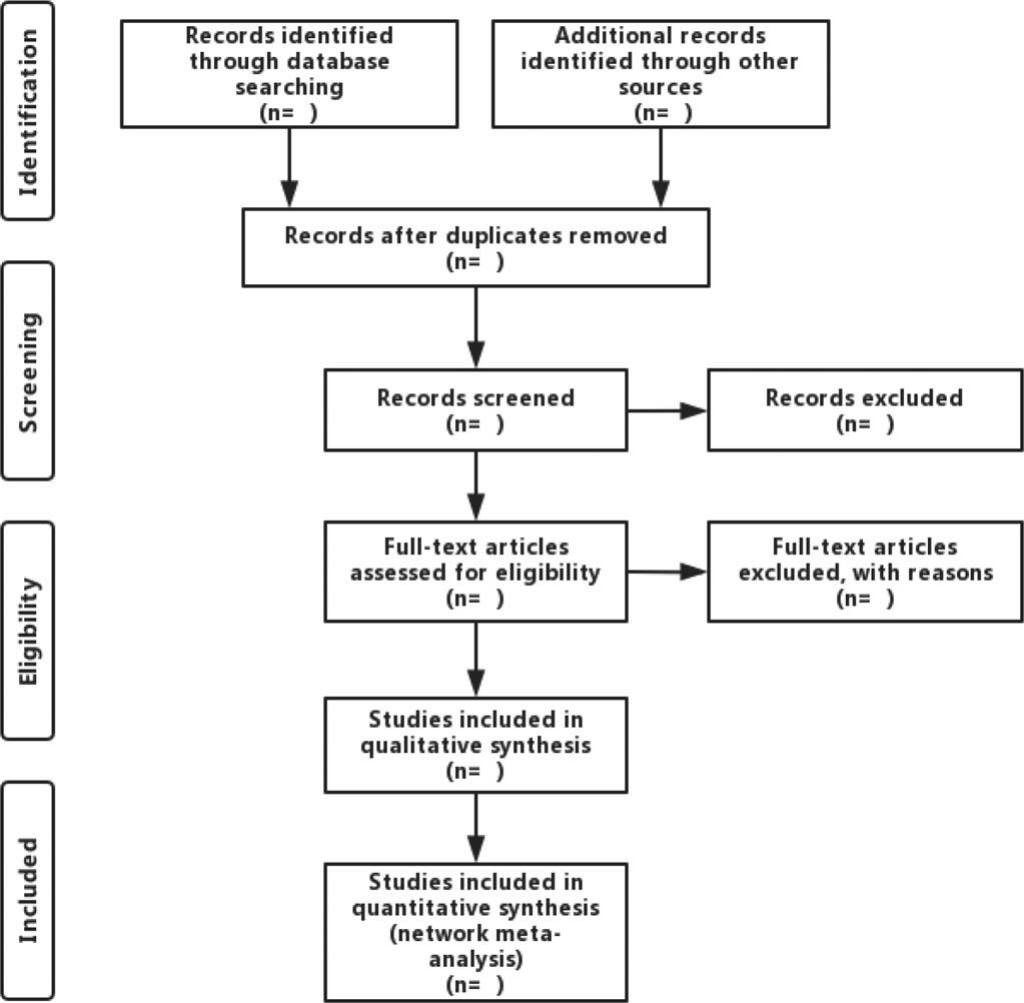
Flow diagram of literature retrieval.

Two researchers will independently assess the titles and abstracts of all studies retrieved through the initial database search, screening them based upon defined inclusion and exclusion criteria. Any discrepancies between these reviewers will be resolved by a third investigator. Two researchers will then independently extract the following data from each eligible study: author names, year of publication, title, average age, gender, study design, participants, total case number, outcomes, and other relevant information. If data are not available, the original authors of the study in question will be contacted when possible. The study screening process is outlined in Figure 1.

### 2.6 Assessment of study quality

Risk of bias for cohort and case–control studies will be assessed using the Newcastle-Ottawa Scale (NOS).^[12]^ The standards of the Agency for Healthcare Research and Quality (AHRQ) will be utilized to assess the methodological of cross-sectional studies.^[13]^ Discrepancies among investigators will be resolved through discussion and consensus, or by a third investigator.

### 2.7 Statistical analyses

Statistical analyses will be conducted using RevMan 5.3.0. The *I*^2^ statistic and Chi-squared tests will be employed to detect heterogeneity, with random-effects models being used for pooled analyses in the presence of high heterogeneity (*I*^2^ > 50%), and fixed-effects models otherwise being used. Odds ratios (ORs) and 95% confidence intervals (CIs) will be employed to analyze dichotomous variables.

### 2.8 Sensitivity analysis

Sensitivity analyses will be performed to confirm the stability of outcome indices.

### 2.9 Subgroup analysis

When sufficient data are available or significant heterogeneity is detected, subgroup analyses will be performed.

### 2.10 Publication bias

When at least 10 studies include data pertaining to a particular endpoint, funnel plots will be used to analyze potential publication bias, which will also be assessed using Egger's test.

### 2.11 Evaluation of evidence quality

The Grading of Recommendations Assessment, Development, and Evaluation (GRADE) method will be used to classify the quality of evidence pertaining to particular outcomes as being high, medium, low, or negligible.^[14]^

## 3 Discussion

Many different variables can influence COVID-19 patient outcomes. The re-positivity phenomenon may be a consequence of SARS-CoV-2 reinfection owing to the insufficient management or clearance of the initial infection.^[15]^ Alternatively, viral shedding from the upper respiratory tract may have ceased in these patients, the virus may have still been present within the lower respiratory tract.^[16,17]^ Older individuals may also be at an elevated risk of COVID-19 re-positivity or reinfection owing to reduced immune functionality, impaired viral clearance, poorer overall health, and other comorbidities.^[18,19]^ It is also possible that some reports of re-positivity may be a consequence of false-positive findings due to technical issues with the associated technologies.^[20]^ We will therefore conduct a systematic analysis to firmly establish the prevalence of SARS-CoV-2 nucleic acid re-positivity among discharged COVID-19 patients and to define risk factors associated with such recurrent viral RNA shedding.

## Author contributions

**Conceptualization:** Yanru Cui, Jilin Wang, Gaofeng Wang, Lizhen Tian.

**Data curation:** Yanru Cui, Jilin Wang, Xiuguo Xie.

**Formal analysis:** Yanru Cui, Jilin Wang, Gaofeng Wang.

**Funding acquisition:** Yanru Cui, Jilin Wang.

**Investigation:** Yanru Cui, Jilin Wang, Gaofeng Wang, Xiuguo Xie, Lizhen Tian.

**Methodology:** Yanru Cui, Jilin Wang, Xiuguo Xie.

**Project administration:** Yanru Cui, Jilin Wang.

**Resources:** Yanru Cui, Jilin Wang, Gaofeng Wang, Xiuguo Xie.

**Software:** Yanru Cui, Jilin Wang, Lizhen Tian.

**Supervision:** Yanru Cui, Jilin Wang, Gaofeng Wang, Xiuguo Xie, Lizhen Tian.

**Validation:** Yanru Cui, Jilin Wang, Gaofeng Wang, Lizhen Tian.

**Visualization:** Yanru Cui, Jilin Wang.

**Writing – original draft:** Yanru Cui, Jilin Wang, Gaofeng Wang, Xiuguo Xie, Lizhen Tian.

**Writing – review & editing:** Yanru Cui, Jilin Wang.
